# Large-scale antibody reactome profiling identifies herpesvirus-autoantigen associations underlying chronic diseases

**DOI:** 10.21203/rs.3.rs-9371123/v1

**Published:** 2026-04-13

**Authors:** Sanghun Lee, Nicole Prince, Qingwen Chen, Xueying Chen, Junwei Lu, Kevin M Mendez, Rachel S Kelly, Julian Hecker, Dmitry Prokopenko, Michael J McGeachie, Rinku Sharma, Yulu Chen, Andrea Aparicio, Tao Guo, Ofer Levy, Nicholas JW Rattray, Zahra Rattray, Christoph Lange, H Benjamin Larman, Jessica A Lasky-Su

**Affiliations:** Channing Division of Network Medicine, Brigham and Women’s Hospital, Boston, MA, USA; Channing Division of Network Medicine, Brigham and Women’s Hospital, Boston, MA, USA; Department of Population Health Sciences, Duke University, Durham, NC, USA; Channing Division of Network Medicine, Brigham and Women’s Hospital, Boston, MA, USA; Department of Biostatistics, Harvard T.H. Chan School of Public Health, Boston, MA, USA; Channing Division of Network Medicine, Brigham and Women’s Hospital, Boston, MA, USA; Channing Division of Network Medicine, Brigham and Women’s Hospital, Boston, MA, USA; Channing Division of Network Medicine, Brigham and Women’s Hospital, Boston, MA, USA; Genetics and Aging Research Unit and the McCance Center for Brain Health, Department of Neurology, Massachusetts General Hospital, Boston, MA, USA; Channing Division of Network Medicine, Brigham and Women’s Hospital, Boston, MA, USA; Channing Division of Network Medicine, Brigham and Women’s Hospital, Boston, MA, USA; Channing Division of Network Medicine, Brigham and Women’s Hospital, Boston, MA, USA; Channing Division of Network Medicine, Brigham and Women’s Hospital, Boston, MA, USA; Channing Division of Network Medicine, Brigham and Women’s Hospital, Boston, MA, USA; Precision Vaccines Program, Department of Pediatrics, Boston Children’s Hospital, Boston, MA, USA; Strathclyde Institute of Pharmacy and Biomedical Sciences, University of Strathclyde, Glasgow G4 0RE, UK; Strathclyde Institute of Pharmacy and Biomedical Sciences, University of Strathclyde, Glasgow G4 0RE, UK; Department of Biostatistics, Harvard T.H. Chan School of Public Health, Boston, MA, USA; Institute for Cell Engineering, Division of Immunology, Department of Pathology, Johns Hopkins School of Medicine, Baltimore, MD, USA; Channing Division of Network Medicine, Brigham and Women’s Hospital, Boston, MA, USA

**Keywords:** Autoimmunity, Autoantibodies, Reactome, Herpesvirus, Chronic disease

## Abstract

Herpesviruses infect nearly all humans and have long been implicated in autoimmune and chronic diseases, yet their immune interactions with host proteins have not been systematically characterized at population scale. We profiled immunoglobulin G reactivities to >4,600 herpesvirus peptides and >15,000 human proteins using multiplexed protein display in two Mass General Brigham Biobank cohorts (discovery n=1,289; replication n=763), with longitudinal electronic health record follow-up. We identified and replicated 3,943 FDR-significant associations across 93 autoantigens, including previously uncharacterized viral–autoantigen axes, such as PHLDA1 and ZNF550. Eleven autoantigens were predicted by viral peptide reactivities with >85% accuracy in independent validation; some exhibited shared viral–host sequence homology, consistent with possible molecular mimicry. Integrating immune reactivities with incident disease outcomes revealed virus-specific network architectures: cytomegalovirus formed the largest multimorbid network, Epstein–Barr virus converged on pleiotropic autoimmune hubs, and herpes simplex viruses formed smaller, partially overlapping autoreactive networks. These findings define a herpesvirus–autoantigen–disease network atlas and prioritize candidate viral–host immune axes for mechanistic investigation.

Autoimmunity involves activation of an immune response against the body’s healthy cells and tissues,^1^ which can damage organs and lead to chronic disease.^2^ Viral infections are recognized environmental triggers of autoimmunity,^3^ potentially provoking aberrant immune responses through mechanisms such as molecular mimicry, bystander activation, and epitope spreading. In some cases, this leads to the development of highly specific autoantibodies that target the host’s own cells and tissues and may ultimately lead to disease development.^7^ These autoantibodies are increasingly recognized as useful biomarkers, particularly during the prodromal period, when cellular damage is occurring in the absence of overt clinical symptoms.^8,9^ Despite substantial progress linking viral exposures to autoantibody formation, these relationships have not been systematically characterized at cohort scale across large, deeply phenotyped populations.

Herpesviruses represent some of the most well-documented viral families implicated in chronic disease, and they have been implicated across a range of conditions.^1,4,10–17^ The *Herpesviridae* family is comprised of three subfamilies based on similarities in virus size and structure: (1) *Alphaherpesvirinae* contains *Simplexvirus* and *Varicellovirus* genera, (2) *Betaherpesvirinae* contains *Cytomegalovirus* and *Roseolovirus* genera, and (3) *Gammaherpesvirinae* contains *Lymphocryptovirus* and *Rhadinovirus* genera.^18^ Herpesviruses are capable of establishing lifelong latency, manipulating host immune regulation, and sustaining chronic immune activation, features that uniquely position them as candidate triggers of autoimmunity.^2,19^ For example, Herpes Simplex Virus 1 and 2 (HSV-1/2) have been associated with development of autoantibodies targeting neurotransmission-related proteins in autoimmune encephalitis.^20^ Epstein-Barr virus (EBV) and Human Herpesvirus 6 and 7 (HHV-6/7) have been linked to neural, synaptic, and glial-associated autoantibodies related to onset of Sjögren’s syndrome.^21,22^ Further, several herpesviruses have known links to cancer development.^23^ Although the *Herpesviridae* family is one of the most well-studied viral groups capable of shaping autoimmunity,^6^ these associations have not yet been systematically characterized across large, deeply-phenotyped cohorts.^24,25^ Across viral species, distinct network architectures emerged: CMV formed the largest and most interconnected disease network, EBV exhibited smaller but highly pleiotropic autoimmune hubs, and the simplex viruses formed compact, partially overlapping networks enriched for respiratory and autoimmune outcomes.

In this study, we conducted a comprehensive, unbiased investigation of viral and autoantigen immunoglobulin G (IgG) “reactomes” using plasma samples from adult participants in the Mass General Brigham Biobank (MGBB). We leveraged Molecular Indexing of Proteins by Self-Assembly (MIPSA) *VirSIGHT^™^* and *HuSIGHT^™^* libraries^26^ to simultaneously profile antibody reactivities to >4,600 herpesvirus-derived tiling 60–amino acid peptide probes, and >15,000 full-length human protein autoantigens. MIPSA is a high-throughput antibody profiling platform enabling large-scale characterization of reactivities to tiled viral peptide probes and full-length human proteins. Using this high-throughput platform, we tested the hypothesis that IgG reactivities to herpesviruses, as markers of prior viral exposures, were associated with distinct autoantigen reactivity profiles. We further evaluated whether these viral-autoantigen reactivity pairs were linked to chronic diseases by integrating electronic health record (EHR) data across >60 diagnoses with up to 30 years of follow-up. Finally, we implemented a network-based analytic approach to characterize the interconnected architecture of herpesvirus exposures, autoantigen reactivities, and diseases. This multidimensional framework provides a hypothesis-generating view of how herpesvirus reactivities, autoantigen responses, and long-term health outcomes may be interconnected in human populations.

## RESULTS

### Summary of Key Findings

**Figure 1:**
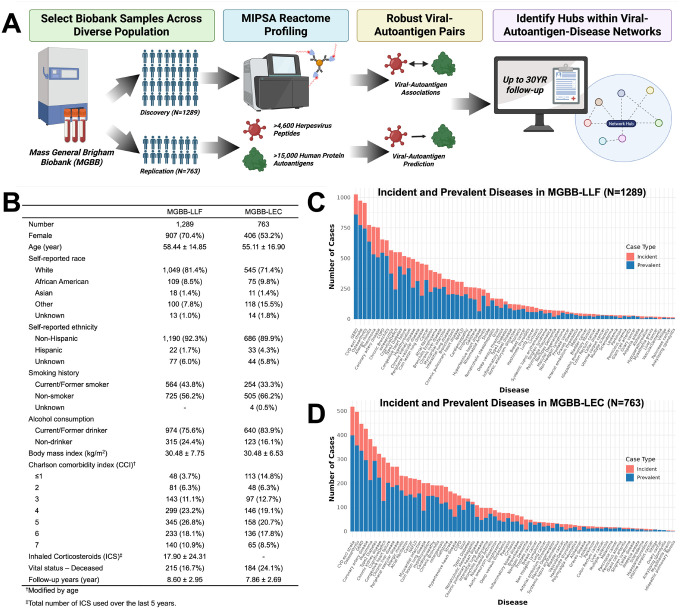
Study Design and Cohort Characteristics. (A) Overview of the study framework. Plasma samples from the Mass General Brigham Biobank were profiled using MIPSA antibody reactome libraries to quantify IgG reactivities to >4,600 herpesvirus peptides and >15,000 human autoantigens. Viral–autoantigen associations were identified in a discovery cohort and evaluated in an independent replication cohort, followed by prediction modeling and integration with longitudinal electronic health record (EHR) data to identify viral–autoantigen–disease networks. (B) Cohort characteristics for the Mass General Brigham Biobank Longitudinal Lung Function discovery cohort (MGBB-LLF, n = 1,289) and the Mass General Brigham Longitudinal EHR Cohort replication cohort (MGBB-LEC, n = 763). (C–D) Distribution of disease diagnoses available in the EHR for MGBB-LLF (C) and MGBB-LEC (D). Bars indicate the number of prevalent and incident cases for each disease outcome across up to 30 years of follow-up. Prevalent cases at the time of blood collection are shown in blue and incident cases occurring after sampling are shown in red.

We profiled antiviral and autoreactive IgG responses in two Mass General Brigham Biobank cohorts using the MIPSA antibody reactome platform and linked these immune signatures to longitudinal clinical outcomes (Figure 1). This analysis revealed widespread viral–autoantigen associations across the human proteome. In the discovery cohort^26^ (MGBB-LLF; n=1,289), we identified 19,270 significant associations between 2,183 herpesvirus peptides representing all nine human herpesvirus species and 992 human protein autoantigens. Of these, 13,312 associations were eligible for replication based on exact peptide-autoantigen matching in the independent cohort, Mass General Brigham Longitudinal EHR Cohort^29^ (MGBB-LEC; n=763), of which 3,943 replicated (29.6%), spanning 881 viral peptides and 93 autoantigens. Among replicated associations, eleven autoantigens were predicted by viral peptide reactivity from HSV-1, EBV, and cytomegalovirus (CMV) with >85% accuracy in independent validation. Sequence homology analyses further identified shared viral–host motifs, including strong similarity between HSV-1 peptides and PHLDA1 and between CMV peptides and ZNF550, consistent with potential molecular mimicry. Network analyses revealed multimorbidity hubs linking herpesvirus exposures to chronic disease outcomes, highlighting distinct virus-specific immune architectures.

### MIPSA profiling identifies replicated herpesvirus–autoantigen associations across two biobank cohorts

**Figure 2:**
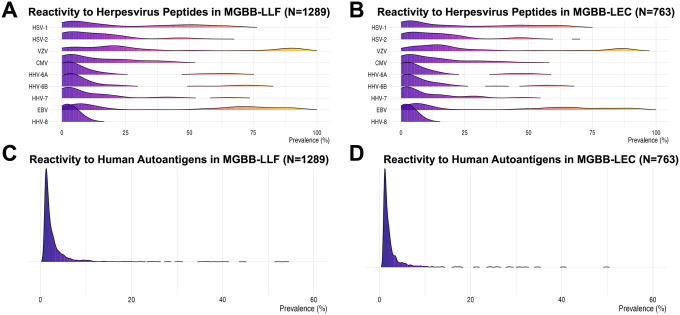
Reactivity to *Herpesviridae* peptides and full-length human protein autoantigens in MGBB-LLF and MGBB-LEC cohorts, as measured by MIPSA. The prevalences of 9 herpesviruses are shown across individual all available viral peptides measured in the MGBB-LLF discovery cohort (n=1289) (A) and MGBB-LEC replication cohort (n=763) (B). Prevalences of reactivities to full-length human protein autoantigens are also displayed for MGBB-LLF (C) and MGBB-LEC (D). For all plots, viral peptides and human proteins are included regardless of inclusion in replication attempt. Abbreviations: Herpes simplex virus 1 (HSV-1); Herpes simplex virus 2 (HSV-2): Varicella Zoster Virus (VZV); Cytomegalovirus (CMV); Human herpesvirus 6A (HHV-6A); Human herpesvirus 6B (HHV-6B); Human herpesvirus 7 (HHV-7); Epstein-Barr Virus (EBV); Human herpesvirus 8 (HHV-8); Mass General Brigham Longitudinal Lung Function (MGBB-LLF); Mass General Brigham Longitudinal EHR Cohort (MGBB-LEC).

Using the MIPSA platform, we detected IgG reactivity to 2,258 of 15,097 (15.0%) human autoantigens and 2,323 of 4,657 (49.9%) *Herpesviridae* peptides at a prevalence of >1% in MGBB-LLF (Figure 1). IgG reactivities were lower in the MGBB-LEC replication cohort, with 1,064 of 15,083 (7.1%) human autoantigens and 2,342 of 4,657 (50.3%) *Herpesviridae* peptides exceeding a prevalence of >1% (Figure 2).

**Figure 3:**
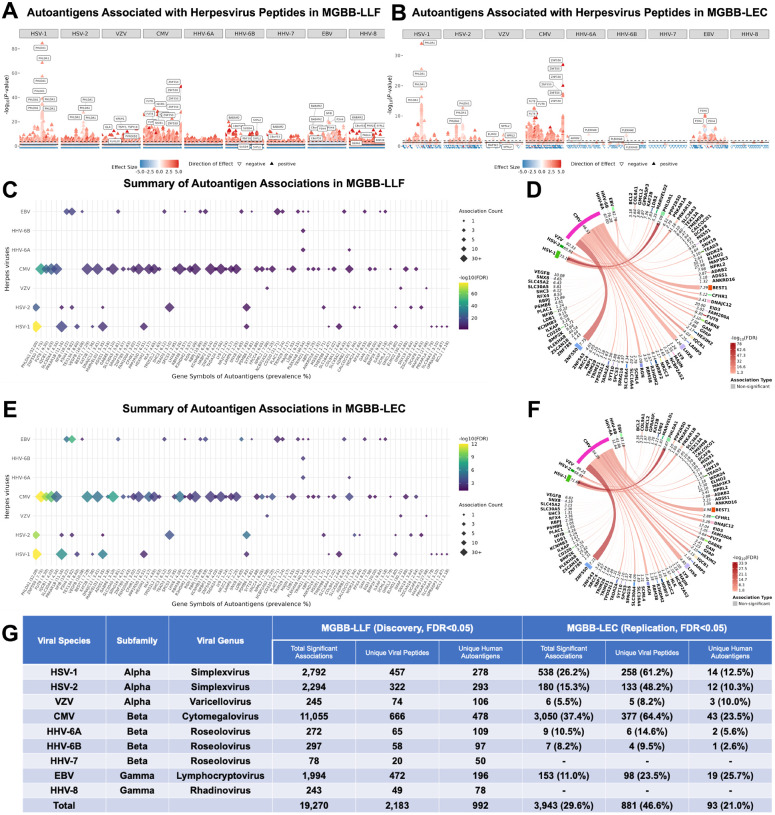
Autoantigen associations with peptides belonging to 9 *Herpesviridae* family viruses. Autoantigen protein targets, denoted by gene symbols, were associated with reactivities to herpesviruses in MGBB-LLF discovery (A) and MGBB-LEC replication (B) cohorts. Significance thresholds are marked by a dotted black line (FDR<0.05) or a solid black line (nominal P<0.05) in (A) and (B). Replicated associations are summarized in diamond plots (C,E) and circular chord diagram plots (D,F), sorted by P-value; the size of each diamond corresponds to the number of individual viral peptides associated with each autoantigen while the color of each diamond indicates the maximum P-value of an individual association between a viral peptide and autoantigen. Models were adjusted for age, sex, body mass index, alcohol consumption, smoking, and Charlson comorbidity index in both cohorts; MGBB-LLF included an additional adjustment for use of inhaled corticosteroid medications. A summary table of herpesviruses and autoantigen associations is provided in (G); unique autoantigens are reported in MGBB-LLF and MGBB-LEC, even if a given autoantigen was associated with multiple herpesvirus peptides and species.

We identified 19,270 viral associations (false discovery rate [FDR]<0.05) between 2,183 herpesvirus peptides and 992 full-length human autoantigens in MGBB-LLF (Figure 3; Supplementary Table 1). These associations spanned all nine human Herpesviridae species, with CMV contributing the largest number of significant viral–autoantigen pairs (11,055 associations involving 666 CMV viral peptides and 478 autoantigens). HSV-1, HSV-2, and EBV also showed broad reactivity landscapes, whereas VZV, HHV-6A, HHV-6B, HHV-7, and HHV-8 contributed fewer associations. Of the 19,270 discovery associations, 13,312 (69.1%) were eligible for replication in MGBB-LEC based on peptide–autoantigen matching and feature prevalence criteria. Because identical viral peptide and full-length protein libraries were used in both cohorts, replication eligibility was determined by feature prevalence rather than differences in library composition. Among these, 3,943 (29.6%) replicated at FDR<0.05 with concordant direction of effect, spanning 881 unique viral peptides and 93 autoantigens. CMV, HSV-1, and HSV-2 showed the highest replication proportions, whereas no associations replicated for HHV-7 or HHV-8 (Figure 3, Supplementary Tables 2–3).

The strongest reactome-wide associations were observed between HSV-1 peptides and PHLDA1, with adjusted P-values ranging from 1.1×10^−78^ to 0.04 (β = 1.4–2.8) in MGBB-LLF and adjusted P-values ranging from 1.6×10^−30^ to 0.04 (β = 0.5–3.4) in MGBB-LEC (Figure 3A–B). Other highly significant replicated associations included HSV-2–PHLDA1, CMV–ZNF550, CMV–FUT8, CMV–IQCB1, EBV–P3H4, HHV-6A/6B–PLEKHA8, and VZV–ELMO2, NPRL2, and MAP3K3 (Figure 3; Supplementary Table 3). Several replicated CMV-associated autoantigens showed particularly large effect sizes in MGBB-LEC (β_max_ > 5), including ZNF550, IQCB1, HEXIM2, and SLC30A4.

Among the 93 replicated autoantigens, CMV was the most broadly-associated virus, with 43 replicated autoantigens, followed by EBV, HSV-1, and HSV-2 with 19, 14, and 12 autoantigens, respectively. Chord diagrams highlighted dense associations for CMV (e.g., ZNF550, DNAJC12, IQCB1, LHX6, and BEST1) and HSV-1 (e.g., PHLDA1 and PRKAR1B), while EBV and HSV-2 links were more diffuse (Figures 3; Supplementary Tables 1–2). Most replicated autoantigens were virus-specific, with the most overlap occurring within genera. Among the 93 replicated autoantigens, 81 (87.1%) were associated with only one herpesvirus species. Shared autoantigen hubs occurred most often between HSV-1 and HSV-2, including GMCL2, LDB2, MARVELD2, PHLDA1, and PRKAR1B, consistent with their close evolutionary relationship and antigenic similarity. Only seven autoantigens showed overlap across viral genera, including PLEKHA8, P3H4, TRIM21, RBM38, ADRB2, TMEM98, and NPRL2. These findings indicate that herpesvirus-associated autoreactivity is largely species(genera)-specific, rather than a single shared autoreactive network.

### Eleven autoantigen reactivities are predicted by three herpesviruses

**Figure 4:**
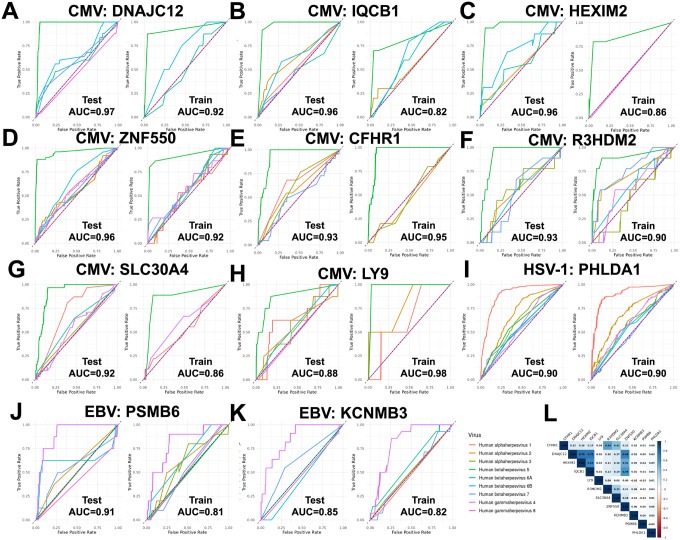
Prediction of autoantigensacross 9 herpesvirus species. Three herpesvirus species predicted reactivities to 11 autoantigens at an AUC>0.85 in the MGBB-LEC test set, including: 8 autoantigens predicted by CMV (A-H); 2 autoantigens predicted by EBV (J,K); and 1 autoantigen predicted by HSV-1 (I). For each viral-autoantigen pair, the left-most panel shows receiver operating characteristic (ROC) curve for prediction by each of the herpesviruses in the MGBB-LLF training set, the second panel shows results for the MGBB-LEC testing set; viral-autoantigen pairs are sorted by viral species, then by descending area-under-the-curve (AUC) value for the MGBB-LEC testing set. A correlation heat map of the fourteen predicted autoantigens across these 3 herpesvirus species is shown in (L) for the MGBB-LLF cohort. Models were adjusted for age, sex, body mass index, alcohol consumption, smoking, and Charlson comorbidity index in both cohorts; MGBB-LLF included an additional adjustment for use of inhaled corticosteroid medications.

We next assessed whether viral peptide reactivities could predict host autoantigen responses. Eleven autoantigens met predefined prediction thresholds, achieving AUC ≥ 0.80 in internal validation (MGBB-LLF test set) and AUC ≥ 0.85 in external validation (MGBB-LEC test set) (Figure 4; Supplementary Figure 1; Supplementary Tables 4–5).

CMV accounted for the majority of highly predictive models, with eight autoantigens predicted at AUC_LEC_>90% (Figure 4A–H). CMV prediction models were enriched for peptides mapping to membrane and glycoprotein functions, and several individual CMV peptides contributed to multiple autoantigen models. For example, a peptide derived from membrane protein RL12 was retained in five prediction models, whereas peptides from glycoprotein O and transmembrane protein UL145 were each retained in four models (Supplementary Table 4). Several CMV-predicted autoantigen responses were also correlated, with the strongest correlation observed between HEXIM2 and DNAJC12 (r = 0.73), partially overlapping immune recognition (Figure 4L).

HSV-1 predicted a single autoantigen response, PHLDA1, with an AUC_LEC_ of 0.90 (Figure 4I). The HSV-1 model included nine peptides spanning tegument, nuclease, ubiquitin ligase, uracil glycosylase, thymidine kinase, nuclear egress, and double-stranded RNA binding functions, indicating that prediction of PHLDA1 reactivity did not depend on a single viral class.

EBV peptides predicted two autoantigen responses, PSMB6 (AUC_LEC_ = 0.91) and KCNMB3 (AUC_LEC_ = 0.85) (Figure 4J–K). Notably, the KCNMB3 model was enriched for latency-associated EBV proteins, including EBNA1 and related latency factors, whereas PSMB6 was predicted by EBV peptides spanning latency (EBNA-2 and EBNA-3C), lytic, and structural functions. Despite these functional similarities, the predictive peptide sets for PSMB6 and KCNMB3 did not overlap

### Sequence homology was observed between herpesvirus peptides and predictive autoantigens

**Figure 5:**
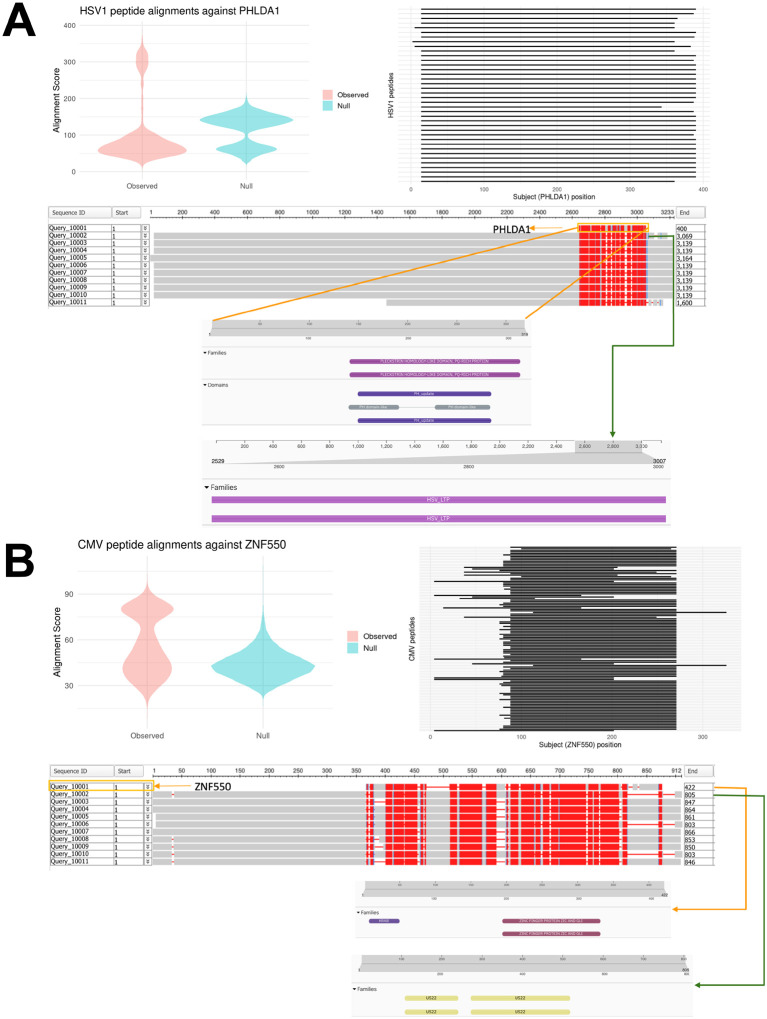
Sequence homology analysis between predicted viral-autoantigen pairs. Sequence alignment was assessed using the Smith-Waterman algorithm in European Molecular Biology Open Software Suite (EMBOSS). Amino acid sequences for pairs of viral peptides and full-length human autoantigens were compared, and alignment scores for each viral peptide against the full-length human autoantigen were compared against an empirical null distribution. HSV-1 peptides and PHLDA1 protein alignments are shown in (A), with additional information on the alignment position range on the autoantigen sequence and conserved domains for peptides with the top 10 alignment scores. CMV peptides and ZNF550 alignments are shown in (B), including plots and visualizations performed for HSV-1 and PHLDA1.

We investigated sequence similarity for all viral–autoantigen pairs exceeding AUC > 0.85 in the MGBB-LEC test set (Supplementary Table 6). The strongest sequence homology signal was observed for HSV-1 and PHLDA1, with a mean Smith–Waterman alignment score of 100.2 (s.d. 83.9) across 39 of 256 associated viral peptides. Relative to an empirical null distribution, 15% of HSV-1 peptides showed significant homology to a conserved PHLDA1 region mapping to pleckstrin homology-like domains. The PQ-rich pleckstrin homology region also showed similarity to HSV-1 viral lipid transfer proteins (Figure 5). A second strong sequence homology signal was observed between CMV and ZNF550, with a mean alignment score of 56.7 (s.d. 19.0). Forty-one percent of associated CMV peptides exhibited sequence homology with ZNF550, including overlap between KRAB domain and Zic/Gli transcription factor motifs with CMV tegument protein US22 (Figure 5). Other viral-autoantigen pairs showed substantially weaker alignment signals (scores <50).

### Herpesvirus–autoantigen networks reveal virus-specific multimorbid disease architectures

**Figure 6:**
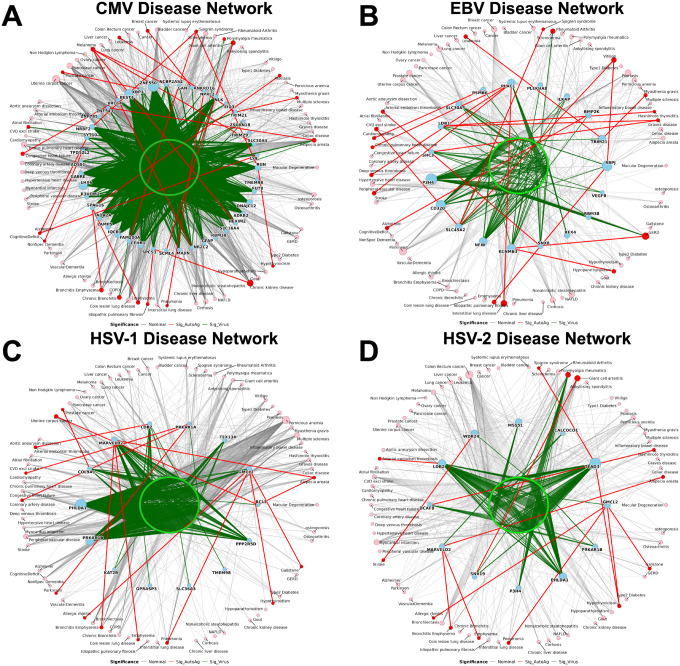
Herpesvirus–autoantigen–disease networks reveal virus-specific multimorbid architectures. Tripartite networks illustrate associations between herpesvirus peptides, autoantigens, and incident disease outcomes. Networks are shown for CMV (A), EBV (B), HSV-1 (C), and HSV-2 (D). Each network highlights virus-specific architectures connecting viral reactivity to multimorbid disease pathways. Nodes represent viral peptides (green), host autoantigens (blue), and diseases (red). The size of autoantigen nodes is proportional to network degree, reflecting the number of disease associations connected to each autoantigen. Thickness of edges between two nodes is relative to the −log_10_(P-value), with smaller P-values showing thicker edges, and autoantigen node size is proportional to the network degree. Edges are color-coded by association type. Red edges denote autoantigen–disease associations and green edges denote viral peptide–autoantigen associations when associations met FDR < 0.05 for the respective comparison. Associations that were nominally significant (P < 0.05) but did not meet FDR significance are shown in grey to provide structural context within the network. Models were adjusted for age, sex, body mass index, alcohol consumption, smoking, and Charlson comorbidity index in both cohorts; MGBB-LLF included an additional adjustment for use of inhaled corticosteroid medication.

To assess clinical relevance, we associated replicated viral peptides and autoantigens with incident disease outcomes in pooled analyses across the MGBB-LLF and MGBB-LEC cohorts. Herpesvirus immune reactivity organized into virus-specific multimorbid disease networks linking viral peptides, host autoantigens, and incident clinical outcomes across multiple disease domains (Figure 6; Supplementary Tables 7–8). Unless otherwise noted, all association findings described below are FDR-adjusted significance for disease incidence. In network visualizations, edges meeting FDR<0.05 are shown in red, whereas nominally significant associations (P<0.05) are displayed in grey to provide structural context. Network visualizations are only shown for viruses with more than one FDR-significant disease association. Comparison of herpesvirus-associated disease networks revealed three distinct architectural patterns: CMV formed the largest multimorbid network centered on autoimmune and oncologic hubs, EBV exhibited fewer but highly pleiotropic autoantigen nodes linking multiple disease domains, and the simplex viruses (HSV-1 and HSV-2) formed smaller but partially overlapping networks enriched for respiratory and autoimmune outcomes.

### Cytomegalovirus (CMV) Network

Among all herpesvirus networks analyzed, CMV formed the largest and most multimorbid architecture, with autoantigen-linked associations spanning 18 diseases across seven clinical domains (Supplementary Table 8). The network was organized around autoimmune and oncologic hubs, with additional respiratory, metabolic/renal, cardiovascular, and neurodegenerative links (Figure 6A).

Autoimmune diseases represented a major axis of CMV-associated reactivity. CMV-linked autoantigens connected viral signals to several autoimmune outcomes, including FAM200A and NRBF2 with Sjögren’s syndrome, RGN with hypothyroidism and Sjögren’s syndrome, SPAG16 with psoriasis, TRIM21 with multiple sclerosis, SCML4 with giant cell arteritis, and ZNF543 with alopecia areata. Several of these nodes also connected to additional disease domains, underscoring the multimorbid structure of the CMV network. The oncologic domain showed the greatest convergence of associations. Melanoma emerged as the most densely connected outcome across all herpesvirus networks, linking CMV reactivity to multiple autoantigens including HEXIM2, SLC30A4, NCBP2AS2, and NR2C2. Additional autoantigens linked CMV reactivity to other malignancies, including TRIM21 with leukemia, ANKRD16 with breast cancer, and XBP1 with prostate cancer, highlighting the breadth of the CMV-associated oncologic footprint.

Respiratory and metabolic/renal disease pathways were also represented. SPCS3 connected chronic kidney disease with idiopathic pulmonary fibrosis, while SPAG16, also linked to emphysema and bronchitis. A direct CMV peptide association with chronic bronchitis further supported respiratory involvement, while NPRL2 was uniquely associated with pneumonia. More limited connections appeared in the neurodegenerative and cardiovascular domains. Three autoantigens linked CMV reactivity to neurodegenerative outcomes, including SYT10 and LY9 with Alzheimer’s disease and TPD52L2 with cognitive deficits. The cardiovascular domain was represented by a single autoantigen association linking XRCC4 to congestive heart failure, with an additional CMV peptide associated with broader cardiovascular disease outcomes.

### Epstein-Barr Virus (EBV) Network

EBV exhibited a disease network characterized by fewer nodes but greater autoantigen pleiotropy, spanning eight clinical domains, the largest number observed for any herpesvirus species in this study. The EBV network consisted of 29 viral peptides and 10 autoantigen nodes linked to 17 outcomes, with several nodes associated with multiple diseases.

Autoimmune diseases again represented a major axis of viral reactivity. EBV-linked autoantigens were associated with Sjögren’s syndrome, vitiligo, hypoparathyroidism, Graves disease, Hashimoto’s thyroiditis, and scleroderma, with several nodes bridging autoimmune outcomes to other disease domains. For example, SLC45A2 was associated with Sjögren’s syndrome and vitiligo and also linked to cardiovascular disease, while RBPJ connected vitiligo with idiopathic pulmonary fibrosis, bridging autoimmune and respiratory pathways. KCNMB3, one of the EBV-predicted autoantigens, linked hypoparathyroidism with gastroesophageal reflux disease, which was also associated with multiple EBV peptides.

Notably, TRIM21 linked EBV reactivity to multiple sclerosis and also showed connections to leukemia, mirroring associations observed in the CMV network and pointing to a conserved viral–autoantigen axis shared across herpesviruses. Other autoimmune-associated nodes were restricted to the autoimmune domain, including LDB1, which encompassed multiple thyroid autoimmune diseases including Graves disease and Hashimoto’s thyroiditis, and SHC3, which was associated with Graves disease. In addition, a single EBV peptide was associated directly with scleroderma.

Beyond autoimmune disease, several EBV-associated nodes linked viral reactivity to cardiovascular and respiratory outcomes. PLAC1 represented the most multimorbid node, connecting EBV signals to peripheral vascular disease, deep venous thrombosis, interstitial lung disease, and chronic pulmonary heart disease. Additional cardiovascular associations included PSMB6 with cardiomyopathy and SNX8 with arterial embolism and thrombosis. A single neurodegenerative association was observed, linking BMP2K to Alzheimer’s disease.

### Herpes Simplex Virus Networks: HSV-1 and HSV-2

HSV-1 and HSV-2 formed partially overlapping disease networks enriched for respiratory and autoimmune outcomes, sharing several autoantigen hubs including GMCL2, MARVELD2, and LDB2. Across both viruses, disease associations were concentrated within the respiratory, autoimmune, and gastrointestinal domains, with the HSV-1 network linking 13 diseases through six autoantigen nodes and the HSV-2 network linking 12 diseases through seven nodes. GMCL2 represented a shared multimorbid hub across both simplex virus networks, connecting viral reactivity to respiratory outcomes including pneumonia and allergic rhinitis, autoimmune disease including hypoparathyroidism, and gastrointestinal disease including gallstones. MARVELD2 similarly spanned multiple domains, linking bronchitis/emphysema with alopecia areata, while LDB2 was associated with arterial embolism and thrombosis.

Beyond these shared nodes, the two simplex virus networks diverged in structure. The HSV-1 network showed a higher density of viral–autoantigen associations and included links to cardiovascular, neurodegenerative, autoimmune, and oncologic outcomes distinct from those observed for HSV-2. PRKAR1A represented a multimorbid hub unique to HSV-1, linking viral reactivity to cardiomyopathy, coronary artery disease, and Parkinson’s disease. Additional HSV-1–specific nodes linked to oncologic outcomes, including COL9A1 with prostate cancer and BCL2 with uterine corpus cancer, with BCL2 also associated with alopecia areata. HSV-1 did not demonstrate direct viral peptide–disease associations, suggesting that disease links within the HSV-1 network may be primarily autoantigen-driven. HSV-2 displayed a different pattern, with associations largely concentrated within autoimmune disease pathways. Autoantigen nodes including TEAD3, SNX19, and DCAF8 were associated with autoimmune conditions such as hypoparathyroidism, Sjögren’s syndrome, giant cell arteritis, and Hashimoto’s thyroiditis. A cluster of highly correlated HSV-2 peptides was also directly associated with polymyalgia rheumatica. The only additional domain represented in the HSV-2 network was cardiovascular disease, with WDR24 linked to stroke.

## DISCUSSION

Despite longstanding evidence linking herpesvirus infections to autoimmune and inflammatory diseases, the immune architecture connecting viral exposure, host autoreactivity, and chronic disease risk remains poorly defined. Here, we provide a population-scale, unbiased characterization of the herpesvirus-autoantigen landscape across all nine herpesvirus species known to infect humans. We combined antibody reactome profiling of viral and host reactivities with decades of longitudinal EHR data, to define a framework linking viral exposures, autoantigen reactivities, and chronic disease. Viral peptides predicted specific autoantigen reactivities with high accuracy, and some viral–autoantigen pairs shared sequence homology, consistent with molecular mimicry one possible mechanism linking herpesvirus exposure and autoantigen reactivity. Importantly, multimorbid autoantigens exhibited broad disease footprints, whereas others showed relative specificity for individual diseases or disease domains, highlighting candidate markers for future study. Together, these findings demonstrate that large-scale IgG reactivity profiling can resolve structured herpesvirus–autoantigen architectures linked to long-term multimorbidity and prioritize virus–autoantigen relationships for mechanistic investigation.

Herpesviruses have been implicated in autoimmune and other chronic diseases for decades^1,4,10–17^, but this work has largely proceeded one disease at a time, without a unifying framework for understanding the broader architecture linking viral exposure, autoreactivity, and clinical outcomes. Our study provides an initial population-scale scaffold for that architecture by identifying reproducible virus–autoantigen–disease networks across multiple chronic disease domains. This type of mapping is a necessary first step for clinical translation: before viral immune signatures can be evaluated as biomarkers or therapeutic targets, the broader landscape of reproducible associations must first be defined. Although our findings do not establish causality and are not yet ready for clinical implementation, they provide a foundation for mechanistic studies and future translational efforts aimed at disease subtyping, risk stratification, and prevention.

A key observation emerging from this work is that herpesvirus reactivities organize around virus-specific autoantigen hubs rather than a single shared autoreactive network. Viral peptides from the same herpesvirus frequently converged on a limited number of host autoantigens, forming distinct virus-centered immune modules. In most cases these hubs were largely specific to individual herpesviruses, suggesting that different viral exposures imprint the immune system through partially independent autoreactive pathways. While cross-virus overlap was limited overall, shared autoantigen hubs were more common between HSV-1 and HSV-2, which is consistent with their close evolutionary relationship and antigenic similarity. This pattern indicates that herpesvirus exposure history may be encoded in virus-specific autoreactive signatures rather than through a single generalized viral autoimmunity axis. Examining the individual herpesvirus networks revealed distinct immune architectures and disease footprints across viral species.

Among the herpesviruses examined, CMV produced the most expansive immune network, with the highest density of viral–autoantigen associations and the largest number of unique associated diseases. CMV reactivities predicted the most host autoantigen responses; several CMV-predicted autoantigens, including HEXIM2, SLC30A4, and LY9, were also associated with higher risk of disease incidence across autoimmune, oncologic, metabolic, and cardiovascular disease domains. This prominent footprint is biologically plausible given CMV’s distinctive immunobiology, which establishes lifelong latency and is known to drive persistent immune activation, clonal expansion of antiviral lymphocytes, and long-term remodeling of the immune repertoire.^34^ Such immune remodeling has been proposed as a mechanism through which CMV influences susceptibility to multiple autoimmune diseases, including SLE, rheumatoid arthritis (RA), systemic sclerosis, T1D, and myasthenia gravis,^39^ underscoring its central role in shaping immune dysfunction. Several CMV-associated autoantigens identified here have established roles in immune and autoimmune disease biology, including TRIM21,^40^ NRBF2,^40^ TRIM29,^41^ SPAG16,^42,43^ and SCML4.^44^ Other targets, including RGN, FAM200A, and ZNF543, have not been widely studied in autoimmune disease and may represent newly prioritized candidates for mechanistic investigation. CMV-associated autoantigens also connected to four autoantigens associated with multiple cancer outcomes, including breast cancer, prostate cancer, melanoma, and leukemia. While XBP1,^45,46^ NR2C2,^47^ and TRIM21^48–51^ have demonstrated mechanistic links to cancer in prior literature, HEXIM2, NCBP2AS2, SLC30A4, and ANKRD16 represent emerging targets that could warrant further study. As the most accurate prediction models in this study were based on CMV peptides, HEXIM2 and NCBP2AS2 may be of particular interest. CMV peptides predicting these autoantigen responses included viral proteins involved in cell membrane biology, glycoprotein processing, and immune evasion—functions central to host–viral interface biology. Taken together, the convergence of CMV-associated autoantigens into disease domains highlights candidate autoantigen hubs for future mechanistic study and suggests potential pathways through which CMV exposure may relate to disease development.

EBV exhibited a distinct immune architecture. Whereas CMV formed a broad network spanning multiple disease domains, EBV reactivities converged on a smaller set of autoantigen hubs enriched for autoimmune and cardiovascular outcomes. However, given the near-universal prevalence of EBV infection in adult populations, the signals observed here likely represent only a subset of the broader EBV-associated immune landscape. The EBV-linked associations identified in this study are nevertheless consistent with longstanding epidemiologic and mechanistic evidence implicating EBV infection in autoimmune diseases including multiple sclerosis (MS),^47^ SLE,^46,52^ RA,^53^ Sjögren’s syndrome,^54^ and cancer.^54^ Within the EBV network, several autoantigen hubs connected antiviral immune responses with multimorbid disease outcomes. PLAC1, TRIM21, LDB1, KCNMB3, RBPJ, and SLC45A2 emerged as central nodes. PLAC1 and RBPJ linked EBV reactivity to cardiovascular and respiratory outcomes, whereas the remaining hubs were primarily associated with autoimmune disease. The presence of TRIM21, a well-established autoantigen in systemic autoimmune disorders, reinforces the biological plausibility of the EBV autoimmune network. In contrast, several other targets—including PLAC1, SNX8, and SLC45A2—have not previously been implicated in cardiovascular or autoimmune outcomes, highlighting potentially novel viral–autoantigen axes for investigation. Notably, EBV-linked autoreactivity to LDB1 was associated with autoimmune thyroid diseases, including Graves disease and Hashimoto thyroiditis. Additional signals included BMP2K, which was associated with Alzheimer disease, as well as several proteins bridging autoimmune and cardiopulmonary outcomes. These patterns suggest that EBV-associated immune responses intersect with a focused set of host pathways that connect antiviral immunity with autoimmune, cardiovascular, and neurologic disease domains.

Predictive modeling further pointed to a potential role for EBV latency biology. Viral peptides predicting host autoreactivity to KCNMB3 were enriched for latency-associated proteins within the EBNA family,^54^ suggesting that immune responses directed against EBV latency programs may contribute to the emergence of specific autoreactive responses. EBNA proteins have previously been implicated in host transcriptional regulation and molecular mimicry, providing a plausible mechanistic framework through which EBV infection could intersect with autoimmune pathways. Although the EBV network detected here appears relatively compact, it likely reflects a broader EBV-driven immune architecture that becomes detectable only when population-scale variation in antiviral immune responses can be resolved.

The immune architectures associated with herpes simplex viruses (HSV-1 and HSV-2) differed from those observed for CMV and EBV. Rather than forming broad networks of host targets, disease associations for the simplex viruses converged on a smaller set of largely shared autoantigen nodes, particularly GMCL2, MARVELD2, and LDB2. These findings suggest that HSV exposures intersect with host immunity through a more restricted set of autoreactive pathways. These multimorbid nodes linked respiratory, gastrointestinal, and autoimmune outcomes, consistent with epidemiologic evidence implicating HSV exposure in autoimmune conditions including multiple sclerosis, rheumatoid arthritis, Crohn’s disease, and psoriasis.^55,56^ Despite this shared architecture, the two viruses exhibited distinct disease signatures. HSV-1 networks highlighted autoantigens associated with oncologic, cardiopulmonary, and neurodegenerative outcomes, including PRKAR1A, BCL2, and COL9A1. PRKAR1A regulates cell growth and signaling pathways,^57–59^ while BCL2 is a well-characterized regulator of apoptosis in cancer biology.^60^ In contrast, HSV-2 reactivities showed a stronger autoimmune footprint, with disease associations converging on TEAD3, DCAF8, and SNX19. These autoantigens have not previously been linked to HSV-2 exposure, although TEAD3 has been implicated in immune regulation,^61^ suggesting that these targets may represent previously unrecognized pathways linking viral infection to autoimmune disease. Together, these findings suggest that simplex virus exposures are associated with a compact autoreactive network architecture, in which a shared core of host targets links HSV-1 and HSV-2 while virus-specific extensions connect these immune signatures to distinct oncologic, neurologic, and autoimmune disease domains.

One of the strongest viral–autoantigen relationships identified in this study involved PHLDA1, particularly within the HSV-1 network. Viral peptides predicted PHLDA1 autoreactivity with high accuracy, and this pair showed among the strongest sequence homology signals observed in the dataset. PHLDA1 is involved in a range of cellular processes—from inflammatory regulation to cell death—and has been implicated in cancer biology, cardiovascular disease, and inflammatory bowel disease.^62^ Interestingly, despite strong viral prediction signals, PHLDA1 did not emerge as a disease-associated hub in either HSV network. This observation highlights an important distinction between viral–autoantigen associations and downstream disease associations: viral peptides may strongly predict autoreactivity to specific host proteins without those responses necessarily mapping onto disease outcomes within the same dataset. Given that PHLDA1 negatively regulates pro-inflammatory cytokine production,^63^ it may nevertheless represent a biologically meaningful immune signature.

In addition to the network architecture itself, predictive modeling further demonstrated that viral peptide reactivities were highly informative of host autoantigen responses. Across multiple viral–autoantigen pairs, patterns of antiviral IgG reactivity predicted specific autoreactive responses with high accuracy, indicating structured immune architectures linking viral exposure history with host autoreactivity. In some pairs, viral peptides and host autoantigens also shared measurable sequence similarity, consistent with the possibility that molecular mimicry may contribute to some of the observed associations. However, the present study does not establish that viral peptides directly induce autoreactive immune responses. Rather, the viral–autoantigen axes identified here represent candidate mimicry relationships that can now be prioritized for targeted mechanistic and experimental investigation.

The network patterns identified in this study may also provide insight into the biological basis of multimorbidity, in which multiple chronic conditions cluster within individuals over time. Several autoantigen hubs connected diseases spanning autoimmune, neurologic, cardiovascular, and oncologic domains, suggesting that shared immune pathways may contribute to susceptibility patterns across seemingly unrelated conditions. In this framework, viral exposures may not act solely as triggers of individual diseases but may instead shape long-term immune remodeling that intersects with multiple disease processes. Viral exposure history may therefore reflect underlying immune trajectories associated with susceptibility to specific constellations of chronic conditions.

These findings also have potential implications for biomarker discovery. Autoantibodies are increasingly recognized as markers of preclinical disease, often detectable years before the onset of clinical symptoms. Although several autoantigens were associated with multiple disease outcomes, the present study was not designed to establish disease specificity relative to healthy controls or diagnostic performance at the individual level; these findings should therefore be interpreted as candidate immune signatures rather than validated clinical biomarkers. The viral–autoantigen networks described here suggest that combined patterns of antiviral reactivity and host autoantigen responses may capture early immune perturbations relevant to multiple disease domains. The atlas presented here provides a framework for prioritizing viral–autoantigen axes for mechanistic follow-up. By narrowing thousands of potential interactions to a smaller set of reproducible immune axes, these results may help guide development of candidate biomarker panels and experimental studies testing whether specific viral peptides can induce autoreactivity.

Leveraging the breadth and heterogeneity of MGBB, we applied MIPSA profiling – a novel, unbiased technology for capturing antibody-binding specificities,^26,70,71^ – to define viral and host reactivities across two cohorts. However, several limitations should be considered. First, viral and autoantigen reactivities were measured at a single time point, precluding determination of the temporal relationship between viral exposure and the emergence of autoreactive responses. Second, the MIPSA platform captures one immunologic axis: serum IgG binding to linear viral peptides and full-length human proteins. It does not comprehensively detect conformational or post-translationally modified epitopes, antibody responses mediated by other isotypes such as IgA or IgM, or compartmentalized immune responses such as mucosal or intrathecal reactivity. In addition, these findings cannot determine the causative role of any autoreactivity as protective or pathogenic. As such, the absence of a detectable signal in this study should not be interpreted as evidence against herpesvirus involvement in disease, as relevant mechanisms may operate through biological pathways not captured by this platform. Third, because our cohorts were not enriched for classic autoimmune disease and the discovery cohort was selected in part for asthma and lung-function phenotyping, disease-specific or genetically restricted virus–autoantigen associations may have been underrepresented. In addition, differences in sample size and cohort composition between the discovery and replication cohorts may have reduced power to detect or confirm associations involving low-prevalence viral reactivities, autoantigen responses, or disease outcomes. Accordingly, absence of a detected association in this study—including previously reported examples—should not be interpreted as absence of biological relevance, as some signals may require autoimmune-enriched cohorts, specific host backgrounds, or targeted disease-focused analyses for detection. Fourth, the high prevalence of certain viral exposures—particularly EBV—may limit the ability to detect the full spectrum of virus-associated autoantigen responses in population cohorts. Fifth, clinical outcomes were derived from EHR records and therefore may be subject to misclassification and differential ascertainment. Finally, although we adjusted for key demographic and clinical variables, including comorbidity burden and inhaled corticosteroid use where available, residual confounding from medication exposures, healthcare utilization, and other unmeasured factors cannot be excluded. In particular, immunosuppressive therapies, antivirals, cancer-directed treatments, and differences in healthcare contact could influence both immune measurements and the likelihood of disease ascertainment in the electronic health record, thereby inflating or attenuating selected associations. In addition, the discovery cohort was enriched for asthma and lung-function phenotyping, which may influence the relative representation of respiratory outcomes. Despite these limitations, the large sample size, replication across independent cohorts, and integration with longitudinal electronic health record data provide a scalable framework for identifying virus–autoantigen relationships relevant to human disease.

In summary, by jointly profiling herpesvirus peptide and human autoantigen reactivities across independent cohorts, we provide a population-scale atlas of herpesvirus–autoantigen relationships. These analyses identify reproducible viral–autoantigen axes and reveal that subsets of host autoantigens function as hubs linking antiviral immune responses with multimorbid disease networks. The resulting immune architecture suggests that herpesvirus exposures may imprint durable, virus-specific autoreactive signatures that connect viral exposure history with susceptibility across multiple disease domains. By prioritizing candidate virus–autoantigen relationships, this work provides a foundation for future studies investigating how antiviral immune responses intersect with autoimmunity and chronic disease biology.

## Methods

### Mass General Brigham Biobank Longitudinal Lung Function (MGBB-LLF) Cohort

The Massachusetts General Brigham Biobank (MGBB-LLF) is a large biorepository that provides access to research data and approximately 130,000 high-quality banked samples (plasma, serum, and DNA) from >165,000 consented patients enrolled in the Mass General Brigham (MGB) medical system, as described previously.^1^ Briefly, patient samples are linked to corresponding Electronic Health Record (EHR) data, dating from the start of their medical history within the MGB network. Data are also derived from surveys administered on lifestyle, environment, and family medical history. The total number of participants from the MGB Biobank who provided plasma samples exceeds 60,000 individuals. Of these, 1289 participants were included in this study for MIPSA profiling based on the presence of at least 3 pulmonary function tests present in the EHR. Samples for the MGBB-LLF cohort were selected as described previously;^2^ briefly, individuals were selected based on all of the following: (1) an asthma diagnosis defined by the asthma prediction algorithm^3^ based on the Research Patient Data Registry (RPDR); (2) a minimum of 3 lung function measures or steroid treatment with matched adrenocorticotropic hormone stimulation testing.

### Massachusetts General Brigham Longitudinal EHR Cohort (MGBB-LEC)

The Massachusetts General Brigham Longitudinal EHR Cohort (MGBB-LEC) was derived from two existing cohorts described in prior publications. The MGB-Aging Biobank Cohort (MGBB-ABC) includes samples from 3,451 randomly selected participants from the MGB Biobank to create a proportionate aging biobank population. Individuals were selected into MGBB-ABC based on age, sex and BMI, and further description is available by Chen et al.^2^ Individuals from MGBB-ABC were combined with additional samples from individuals in the in the MGBB derived from the Longitudinal Electronic Omics of COVID-19 Cohort (LEOCC);^3^ notably, all samples utilized in this study were selected prior to the COVID-19 pandemic, so no LEOCC individuals had experienced a COVID-19 infection at the time of sampling used for the present study. A total of 300 individuals were included from MGBB-ABC and a total of 463 individuals from LEOCC were included, for a total of 763 samples in the newly-derived MGBB-LEC cohort. Blood samples were collected either as part of clinical care or through research draws at Mass General Brigham clinical sites; these were used for serum, plasma, and DNA/genomic research. Each blood draw typically involved collecting 30–50 ml of blood, which was linked to the corresponding clinical data from the EHR. Comprehensive EHR, metabolomic profiling, proteomic profiling, and epigenetics are available for select subjects in MGBB, and 300 samples with MIPSA profiling were included in this study based on availability of clinical lab data in the EHR.

### Clinical Outcome Definitions and Covariates for MGBB-LLF and MGBB-LEC Cohorts

The MGB Phenotype Discovery Center (PDC) integrates multiple data sources, including the Research Patient Data Registry (RPDR), health information surveys, and genotype data, into a centralized Biobank Portal. This portal combines specimen and EHR data into a comprehensive SQL Server database with a user-friendly web-based application.^4^ The portal incorporates phenotyping approaches implemented through the Integrating Biology and the Bedside (i2b2) toolkit.^5^ These clinical phenotypes were defined through the implementation of multiple validated phenotyping algorithms that incorporate a range of information, including structured EHR data, narrative clinic notes, and participant-reported survey data.

We defined 73 disease diagnoses using the following framework. For outcomes with available validated phenotyping algorithms, we assigned disease status according to those validated definitions, where the date of disease onset corresponded to the earliest qualifying ICD9 or ICD10 diagnosis date. For clinical outcomes without an available a validated phenotyping algorithm, disease status was defined by the presence of at least two ICD-9 or ICD-10 diagnosis codes in the EHR, and the date of initial disease was assigned as the date of the first qualifying diagnosis code. Prevalent disease was defined based on qualifying diagnoses recorded on or before the date of blood draw for MIPSA profiling, whereas incident disease was defined using first qualifying diagnoses occurring after blood draw. This framework was applied across all 73 diseases analyzed.

At the time of enrollment into MGBB, participants completed questionnaires capturing demographic characteristics, lifestyle factors, and basic health history. Covariate information used in the analyses was obtained from these questionnaires and linked EHR data, including the following: age (years), biological sex (male vs. female), body mass index (BMI), race (white vs. non-white), alcohol consumption (no alcohol use, former alcohol use, current alcohol use), smoking (never smoker, former smoker, current smoker), total number of inhaled corticosteroid (ICS) used over the last 5 years as measured from the RxNorm prescription database, and the Charlson comorbidity index (CCI). To assess comorbidity burden independent of age, we derived a modified CCI by removing the age-weighted points from the standard calculated index. The original CCI assigns additional points for increasing age (1 point for ages 50–59, 2 points for 60–69, 3 points for 70–79, 4 points for 80–89, and 5 points for ≥90 years).

### Molecular Indexing of Proteins by Self-Assembly (MIPSA)

MIPSA antibody reactome profiling was performed using plasma samples from the MGBB-LLF and MGBB-LEC cohorts by Infinity Bio, Inc., as described previously.^6^ MIPSA libraries comprise self-assembled protein-DNA conjugates that represent comprehensive antigen libraries covering viruses (the VirSIGHT library) and the human proteome (the HuSIGHT library). MIPSA peptide libraries are designed by first clustering input protein sequences at the genus level (~2M sequences from all known human-infecting viral taxa for VirSIGHT, and all ~385,000 human protein sequences in UniProt and RefSeq for HuSIGHT), and then creating multiple sequence alignments (MSAs) for each cluster. Peptide tiles (~60aa for VirSIGHT and ~90aa for HuSIGHT) are designed to step across each MSA (40aa step size for VirSIGHT and 60aa step size for HuSIGHT). Finally, redundant tiles are removed and the peptides are reverse translated using an E. coli codon usage preference table. Oligonucleotide libraries encoding the peptides are synthesized by Twist Bioscience and cloned into the MIPSA expression vector along with random, base-balanced DNA barcodes. The VirSIGHT peptide library comprises 285,669 viral peptides covering 529 viral species, while the HuSIGHT peptide library comprises 353,034 human peptides, including 880 mature chain secreted human protein isoforms. In this study, the HuSIGHT peptide library was mixed with a MIPSA library comprising 16,516 full-length human proteins. In the text, HuSIGHT refers to the combination of the human peptide and human full-length MIPSA libraries. The viral peptide and full-length protein libraries were identical across the discovery and replication cohorts; replication eligibility was therefore determined by feature prevalence rather than library overlap.

MIPSA libraries are separately incubated with 0.1 ml of each donor’s plasma, before protein A and protein G coated magnetic beads (Dynabeads, Thermo Fisher Scientific) are added to capture the IgG antibodies and IgG-bound MIPSA library members. Immunocaptured MIPSA library members are identified by PCR amplification and sequencing of their uniquely-associated DNA barcodes. Illumina sequencing generates FASTQ files for each plasma sample and positive and negative controls that are run alongside the samples on each 96-well plate. FASTQ samples are aligned using Bowtie2 and compared to controls using EdgeR for data normalization and fold-over-background estimation. A MIPSA library member is considered reactive (“a hit”) when its FDR-adjusted P-value (using the Benjamini-Hochberg procedure of false discovery rate adjustment) of comparison against a set of “mock IPs” (no plasma input) is less than 0.05 for peptides and less than 0.001 and the fold over background value is greater than 2 for full-length proteins. Because the full-length human protein library represents a substantially larger hypothesis space than the viral peptide library, a more stringent statistical threshold (FDR < 0.001) together with a fold-change requirement (>2 over background) was applied to limit false-positive autoantigen calls.

Additional details of MIPSA antibody reactome profiling can be found in the Supplementary Information. For human autoantigen reactivities, HuSIGHT peptide reactivities are summarized at the protein level by the maximum reactive peptide. In this study, analysis of binarized MIPSA viral and autoantigen reactivities (hits) at the protein level were used for associations between viral peptides and autoantigens while continuous reactivity values were used for associations with viral peptides and autoantigens with disease outcomes.

### Statistical Analysis

We evaluated pairwise associations between human autoantigen reactivity (HuSIGHT hits) and viral peptide reactivity (VirSIGHT hits) using multivariable logistic regression. For every unique autoantigen–viral peptide combination, we fit a generalized linear model with a binomial error distribution and logit link using glm() in R, treating autoantigen status (hit = 1, non-hit = 0) as the dependent variable and viral-peptide status (hit = 1, non-hit = 0) as the primary predictor. Models were run separately for the nine *Herpesviridae* species assayed by MIPSA: Human alphaherpesvirus 1 (HSV-1), Human alphaherpesvirus 2 (HSV-2), Human alphaherpesvirus 3/Varicella zoster virus (VZV), Human betaherpesvirus 5/cytomegalovirus (CMV), Human betaherpesvirus 6A (HHV-6A), Human betaherpesvirus 6B (HHV-6B), Human betaherpesvirus 7 (HHV-7), Human gammaherpesvirus 4/Epstein-Barr virus (EBV), and Human gammaherpesvirus 8 (HHV-8).

To reduce sparsity, we required a prevalence ≥ 1 % for both VirSIGHT peptide hits and HuSIGHT protein hits before inclusion in any model. All regressions were adjusted for potential confounders measured at blood collection as described in more detail above: age, biological sex, BMI, race, alcohol consumption, smoking, Charlson comorbidity index, and total number of inhaled corticosteroid used over the last 5 years. Multiple-testing control in the discovery (MGBB-LLF) cohort was performed with the Benjamini–Hochberg procedure, maintaining a false-discovery rate (FDR) of 5%. Associations passing this threshold were taken forward for replication in the independent MGBB-LEC cohort, where significance was declared at adjusted P-value<0.05 after the same covariate adjustment. Associations were considered replicated in MGBB-LEC based on the following criteria: (i) viral-autoantigen associations met an FDR-adjusted P-value<0.05 threshold, (ii) viral-autoantigen associations were in the same direction of effect as MGBB-LLF, and (iii) viral peptide MIPSA IDs and autoantigen MIPSA IDs pairs were an exact match to identifiers in MGBB-LLF.

All autoantigens and viral peptides that met the discovery-stage FDR threshold in the MGBB-LLF cohort and replicated in the MGBB-LEC cohort were subsequently evaluated for association with clinically ascertained diseases. To maximize statistical power given the rarity of specific diseases and reactive signals, data from the MGBB-LLF (n = 1,289) and MGBB-LEC (n = 763) cohorts were pooled for these analyses. The primary predictor was the immune marker level, coded as a continuous reactivity value for both HuSIGHT proteins and VirSIGHT peptides. The outcome was defined based on the analysis type:

1. Cox Regression (Survival): The dependent variable was the time to incident diagnosis. was the dependent variable,
2. Logistic Regression (Case-Control): The dependent variable was binary disease status (Case = 1, Control = 0). Individuals with a recorded diagnosis were defined as cases, while those without a diagnosis at any time point served as controls.

The primary analysis used Cox Proportional Hazards Regression with incident disease associations. A secondary analysis used Logistic Regression to assess prevalent disease associations and is provided in the supplemental material only. To ensure robust estimation in the presence of rare signals or unbalanced groups, we employed a dynamic model selection algorithm based on data sparsity. For each MIPSA reactivity-disease pair, we constructed a 2 × 2 contingency table based on MIPSA positivity (signal > 1) and disease status. If any cell in the contingency table fewer than 5 observations, we utilized Firth’s penalized likelihood method. This approach reduces small-sample bias and manages potential separation issues. We utilized the logistf package (v1.26.1 in R) for penalized logistic models and the coxphf package (v1.13.4 in R) for penalized Cox models. For variables satisfying the sample size threshold (n ≥ 5 per cell), we employed standard maximum likelihood estimation (standard GLM or standard Cox proportional hazards via the survival package). All models were adjusted for the covariates defined in the pairwise analysis All models were adjusted for the covariates defined in the pairwise analysis between autoantigens and viral peptides. Model fit was assessed using the Akaike Information Criterion (AIC). All analyses were performed using R statistical software.

### Networks of Viral Peptides, Autoantigens, and Diseases

We constructed tripartite association networks for Herpesviridae species showing significant reactivity by integrating results from multivariable Cox (incidence) regression analyses. To visualize the hierarchy of associations, nodes were organized into a custom concentric layout comprising three distinct layers: (i) an inner core of viral peptides, annotated with the UniProt accession codes of the corresponding VirSIGHT peptide; (ii) a middle ring of autoantigens, annotated with HGNC gene symbols; and (iii) an outer ring of diseases. To facilitate clinical interpretation, disease nodes in the outer ring were spatially grouped by clinical category. Edges connecting viral peptides to autoantigens represent associations that replicated in the MGBB-LEC cohort, while edges linking immune markers (viral peptides or autoantigens) to diseases denote associations identified in the pooled MGBB-LLF and MGBB-LEC models. To distinguish robust signals, edges satisfying an FDR threshold of < 0.05 (“Significant”) were visually emphasized with distinct coloring with edges where a green edge indicates a FDR significant viral-autoantigen association and a red edge indicated a FDR significant immune marker-disease association. To provide more context of the overall network structure, nominally significant immune marker associations, where P<0.05 but did not meet the FDR <0.05 threshold, were rendered as thin grey edges (“Nominal”). Node sizes for autoantigens and diseases were scaled proportionally to their degree (the total number of significant connections), to highlight highly connected nodes, while viral peptide nodes were rendered at a fixed size. Each network was visualized as a directed graph using the Fruchterman–Reingold force-directed layout, rendered with the igraph (v2.1.1 in R) and ggraph (v2.2.1 in R) packages.

A Sankey-style summary model was used to illustrate the multimorbid relationship between herpesvirus, multimorbid hub autoantigens, and disease domains. Disease diagnoses were categorized 10 disease domains via ICD-10 ontology and clinical groupings. Edge width was scaled proportional to −log10(P-value) to convey statistical strength of the association the autoantigen node size is proportion to the number of significant viral peptides, and the disease domain node size is proportional to the total number of autoantigen-disease associations within that disease domain.

### Prediction Models

For each *Herpesviridae* species exhibiting replicated viral-autoantigen associations, we concatenated the binary viral-peptide hit matrix with the corresponding autoantigen vector. A separate LASSO logistic-regression model was trained for each target autoantigen, treating the full set of virus’s peptides as candidate predictors. Models were fitted with glmnet package (v4.1–10 in R) and tuned by five-fold cross-validation (cv.glmnet in R, alpha = 1); the regularization parameter λ that minimized the mean cross-validated deviance (λ_min_) was selected. Peptides with non-zero coefficients at λ_min_ defined the virus-specific peptide signature.

The fitted models were evaluated on the MGBB-LEC cohort (external test set) and a held-out subset of the MGBB-LLF discovery cohort (internal validation). Discriminative performance was quantified using the Area Under the Receiver Operating Characteristic (ROC) Curve (AUC) (pROC package v1.19.0.1). Viral-autoantigen combinations were retained for further analysis only if they achieved an AUC ≥ 0.85 in the external test set and an AUC ≥ 0.80 in the internal validation set. To obtain coefficient statistics for the selected features, we refitted ordinary least-squares (OLS) regression models using only the LASSO-selected peptides. This unpenalized approach provided coefficient standard errors and two-sided t-test P-values. Furthermore, individual viral peptides were evaluated using 2 × 2 contingency tables (autoantigen presence × peptide presence) and χ test. We calculated sensitivity, specificity, and balanced accuracy (defined as [sensitivity + specificity]/2). Peptides were ranked balanced accuracy and visualized in scatter plots of sensitivity versus specificity, with points colored according to their AUC values.

### Sequence Similarity Analysis

Amino acid sequences of viral peptides and autoantigens were compared for viral-autoantigen pairs identified in association analyses. All associated viral peptides were compared to the full-length autoantigen sequence using the Smith-Waterman local alignment implementation from The European Molecular Biology Open Software Suite (EMBOSS) package.^7^ Sequences of autoantigens were obtained from UniProt and GenBank.^8^ The BLOSUM62 scoring matrix was used in the local alignment mode to score similarities between amino acids and identify sequences with potential homology. Other parameters were left at default settings (e.g., gapopen 10, -gapextend 0.5). We randomly permuted the amino acids in each subject sequence (human autoantigens) 1000 times (seed = 123) and used the permuted sequences to conduct sequence alignments. Each viral peptide was then aligned against the 1000 permuted human peptides to generate a null alignment score distribution. Empirical null distributions based on the Smith-Waterman alignment scores were then computed using the ecdf function in R. We computed the quantiles of each observed alignment score against the empirical null and corresponding one-sided P-values. Alignments exceeding the 95% quantile were considered extreme to the empirical null distributions. Top pairs were to assess aligned regions. The viral and autoantigen sequences were aligned and plotted together using the Constraint-based Multiple Alignment Tool (COBALT)^9^ and visualized using the NCBI Multiple Sequence Alignment Viewer version 1.26.0.^10^ The COBALT result visualizations were colored using the conservation palette for positions without gaps, where highly conserved and less conserved amino acid positions were defined by the relative entropy threshold of the residue. Positions colored in red indicate high conservation and positions colored in blue indicate lower conservation. InterPro^11^ was used to identify the families and domains of the highly conserved regions for both viral and human peptides found in COBALT.

## Supplementary Material

Supplementary Files

This is a list of supplementary files associated with this preprint. Click to download.

- SUBMITCLEANSIViralHumanReactivities.docx
- SupplementaryTable2LLFviralauto.xlsx
- SupplementaryTable3LECReplicated.xlsx
- SupplementaryTable4allpred.xlsx
- SupplementaryTable5predpeptides.xlsx
- SupplementaryTable7viraldisease.xlsx
- SupplementaryTable8autodisease.xlsx
- SUBMITCLEANSIViralHumanReactivities.docx

## Data Availability

Processed MIPSA viral peptide and full-length autoantigen reactivity data underlying this study will be deposited in the NIH ImmPort (Immunology Database and Analysis Portal) repository to ensure public accessibility. Deposited datasets will include normalized reactivity values, statistical association metrics, and relevant annotation information for viral and autoantigen features. De-identified, limited clinical metadata associated with study participants will be made available to the extent permitted by Mass General Brigham Biobank participant consent and institutional review board policies. Individual-level data requiring controlled access may be requested through the Mass General Brigham Biobank under standard data use agreements. Data Use Agreement with the corresponding author (Jessica Lasky-Su) by email (rejas@channing.harvard.edu).

## Code availability

All R scripts used to develop and validate viral–autoantigen associations, network models, and predictive algorithms are available at the Lasky-Su Lab GitHub repository: https://github.com/LaskySuLab/MIPSA-HSV The proprietary MIPSA bioinformatics pipeline for antibody reactome profiling was implemented by Infinity Bio, Inc. according to standardized protocols. Access to this pipeline can be requested under a material transfer or collaboration agreement with Infinity Bio.
